# Foresight in the time of COVID-19

**DOI:** 10.1016/j.lanwpc.2020.100049

**Published:** 2020-12-09

**Authors:** Maria Isabella Gariboldi, Vivian Lin, Jessica Bland, Mallika Auplish, Amy Cawthorne

**Affiliations:** a Data, Strategy and Innovation, World Health Organization (WHO), Western Pacific Regional Office (WPRO), 669 Ermita Manila 1000, Metro Manila, Philippines; b Public Health, LKS Faculty of Medicine, University of Hong Kong, 21 Sassoon Rd, Pok Fu Lam, Hong Kong; c School of International Futures, 49 Brick Ln, Spitalfields, London E1 6PU, United Kingdom

**Keywords:** Health policy, COVID-19, Foresight, Pandemic response, Futures thinking

## Abstract

Foresight methodologies enable individuals and organizations to envision different future scenarios and plan for greater future resilience. However, foresight is an underused methodology in the Western Pacific region for health policy development that could be extremely beneficial, among other areas, in the context of public health emergency response. We present lessons learned from the application of foresight methodologies through remote, agile think tank sprints to inform the World Health Organization (WHO) Western Pacific Regional Office's (WPRO) response to the COVID-19 pandemic. Four think tanks were set up in topic areas of interest. The think tanks used a six-step foresight methodology to develop scenarios for the pandemic in an 18-month horizon. Backcasting was used to generate recommendations for WHO response and support for countries. This case study demonstrates the value of using foresight methodologies in public health, and specifically in the context of a public health emergency, and its ability to inform more context-appropriate and future-proof responses.

**Funding:**

Japan.

## 1 Introduction

A pandemic response is, by necessity, driven by the day-to-day “firefighting” and the need to keep up to date with a constant stream of evolving information. Public health emergency management generally focuses on the implementation of organizational and programmatic standards and incident management systems [1]. A pandemic like COVID-19, however, is not an acute event. Beyond the immediate shock to health and health systems, the pandemic will inevitably have longer-term consequences on countries’ social, technological, economic, environmental and political landscapes [2,3], as well as indirect impacts on health [4], [5], [6], [7], [8], [9]. These landscapes will affect the vulnerability of countries, and specific populations within countries, to the short- and long-term effects of the virus. Different countries have also employed different strategies [10,11], resulting in divergent, and not yet predictable, futures unfolding, ranging from health systems collapse to the pandemic acting as a catalyst for modernizing health systems. Even should a preferred scenario unfold, countries will be faced with new questions and challenges, as evidenced, for example, by the increasing controversy surrounding data privacy [12] as technologies are harnessed by fragmented stakeholders in the fight against the virus.

The COVID-19 pandemic has resulted in an unprecedented situation for the World Health Organization (WHO) in terms of its need to tailor its approach to countries that are responding and experiencing consequences of the pandemic differently. In order to support countries in managing this rapid and diverse change, the organization needs to be adaptive and responsive. Considering the need to prepare for the worst scenarios and proactively build towards preferred ones, the Western Pacific Regional Office (WPRO) set up foresight think tanks implementing a multi-disciplinary approach to envisioning possible futures and inform a strategic response to the pandemic. The goal of this article is to document WPRO's experience in using foresight to plan for the needs of the possible “new futures” emerging from the pandemic and make a case for the use of foresight in health policy more broadly. The following section provides background on the use of foresight in public health and current barriers to its implementation. We then describe WPRO's application of foresight to complement traditional pandemic response systems in the context of COVID-19. Finally, we present reflections emerging from the experience on the benefits of foresight in pandemic response and health policy and planning more broadly.

## 2 Foresight in public health

Conventional health system and program planning are often based on a rationalist and evidence-based model that assumes control and thus does not appropriately account for influences of factors external to the health system [13]. This can result in short-sighted health policies that may not meet the longer-term challenges of non-linear changes or radical systemic disruptions, as are emerging from the COVID-19 pandemic.

Foresight is an effective tool in preparing for the future when linear thinking is inadequate and uncertainties prevail. While forecasting strives to generate predictions of the near future based on past trends [14], foresight comprises a range of methodologies that aim to map drivers of change, trends and areas of uncertainty usually in a 20–50 year horizon [15]. Policy makers can use these tools to produce scenarios, build a common vision, and develop strategies to mitigate risks, be resilient to uncertainties and leverage emerging opportunities [15]. Backcasting is a methodology commonly used in foresight by which a plan is developed working back from a future vision.

Policy makers and governments can benefit from futures thinking to anticipate future challenges and build resilient strategies [16]. The UK Government Foresight Programme, for example, has employed a foresight approach to tackling obesity [17]. The project involved visualizing scenarios for obesity in 2050 to build a sustained response. The Dutch Public Health Foresight Study (PHFS) 2018 was used to develop the National Health Policy Memorandum. Youth mental health, an issue largely based on qualitative analyses, emerged unexpectedly as one of the salient themes, demonstrating the importance of integrating qualitative and quantitative approaches [18]. Several non-governmental and multi-lateral organizations have conducted foresight projects in the Asia-Pacific region. For example, the United Nations Development Programme (UNDP) was involved in the development of *Foresight eXplorer*, a card game that helped participants envision values, behaviours and structures for Tonga's future [19]. The Asian Development Bank (ADB) facilitated a foresight workshop in Cambodia in 2019, which covered topics including women's empowerment and technology in governance [15].

In the context of COVID-19, initial applications of foresight to envision the “new future” are emerging. Inayatullah and Black [20] argue that COVID-19 is not a *black swan* (or *wild card*) event – a high impact, low probability or unexpected event – and articulate why it had been anticipated by people working on emerging infectious diseases. The authors further provide four brief future scenarios to illustrate distinct metaphors at work in public commentary about how the pandemic will change life in the future: *Zombie Apocalypse, the Needed Pause, Global Health Awakening* and *the Great Despair*. These are given as a jumping off point for others developing alternative futures.

Foresight has been successfully applied in private sector companies, Shell scenarios in the 1970s [21] being a particularly famous example having had a transformative influence on a company. “Firms”, however, have the advantage of having clearer boundaries and goals, enabling them to derive real benefits from foresight more immediately. Rohrbeck and Kum [22], for example, assessed the impact of future preparedness in 2008 on firm performance, including profitability and market capitalization growth. Some of the barriers resulting in infrequent use of foresight methodologies in public health include short policy timeframes and rigid and compartmentalized organizational cultures and structures. Public health policy also traditionally relies on quantitative data and models, such as supply and demand models for health workforce planning [14]. Implementing strategic foresight in the public sector also has the unique challenges of addressing divergent and contested objectives across multiple spheres, including the economy, society and the environment, while having to also manage a much more complex set of stakeholder interests. Last, Vollmar and colleagues [23] point out that foresight methodologies are not standardized and often poorly reported, which may contribute to their limited popularity in health policy and planning.

## 3 Implementation of foresight in WPRO's response to COVID-19

**Fig. 1:**
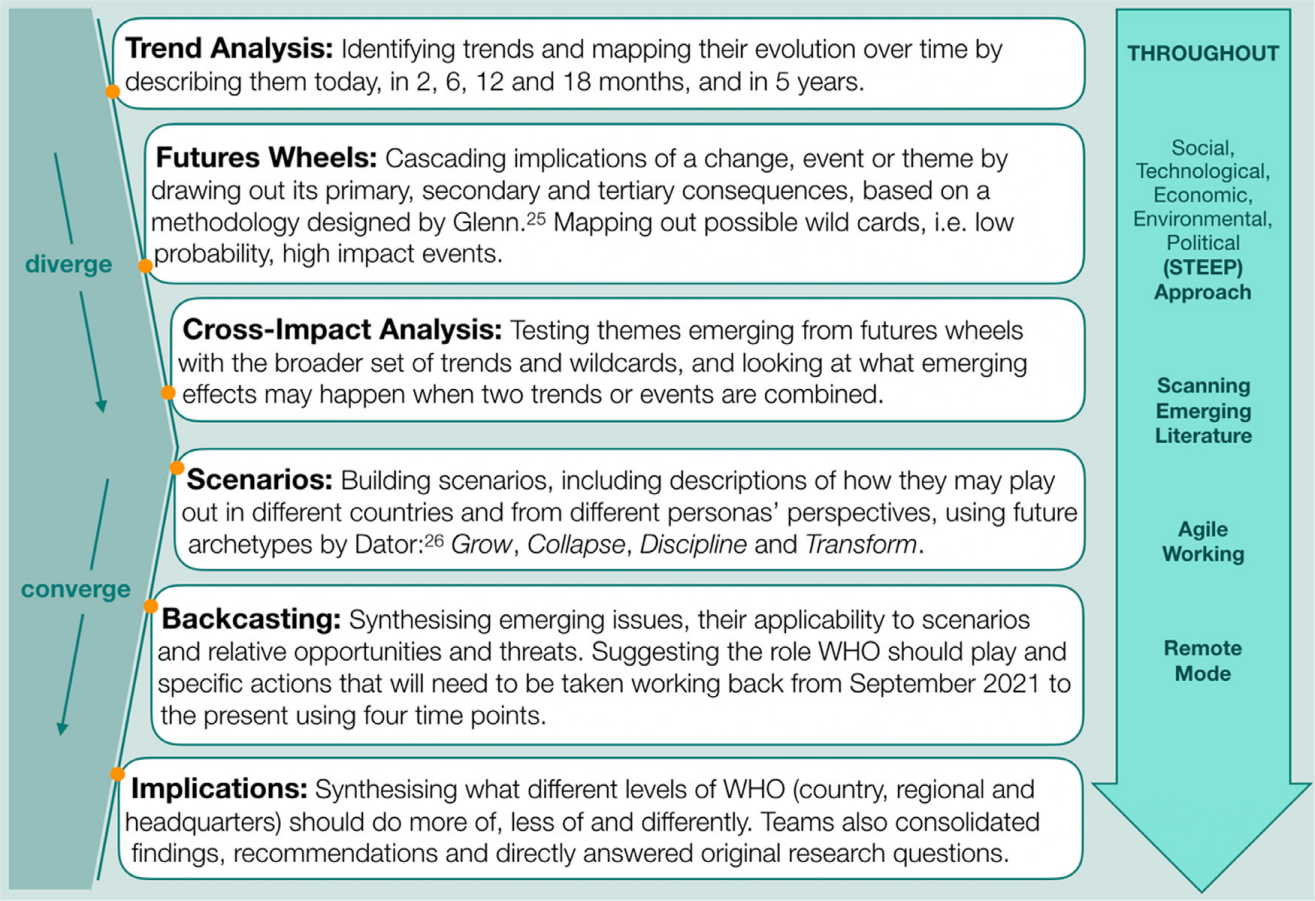
**Summary of the methodology employed by each think tank team.** The six steps are shown as well as cross-cutting themes. The process can loosely be divided into a divergent and a convergent thinking phase [25,26].

In 2020, WPRO began to implement the new Regional Director's vision [24], which asked Member States to take a long-range view and for WHO to develop backcasting as a new way of working. As part of the COVID-19 response, four agile teams were established to bring together WPRO staff and external academics over the course of several weeks from March to June 2020 to produce recommendations for WHO based on futures methods around four themes: equity, non-pharmaceutical interventions (also known as public health and social measures, or as community mitigation measures), non-COVID mortality and ethics. A total of 34 people participated in the think tanks, among which 13 were external to WHO. To assemble diverse teams, a team lead was identified for each think tank theme and was asked to select participants for his/her think tank. Both WHO and external participants were invited based on their technical expertise, skillsets and interest in the think tank theme. External participants were largely from within the Western Pacific region. An 18-month horizon was chosen for foresight exercises based on vaccine development forecasts. The think tank teams served two goals: a strategic one of integrating futures into WPRO's strategic planning for COVID-19 response by surfacing opportunities and risks beyond the most immediate issues, and an organizational development one of fostering mindsets for futures thinking in routine work. Each team was composed of 6 - 13 people. Facilitation guides and exercises were provided by the School of International Futures (SOIF) based on the methodology devised by WPRO to support country health systems transformation. Sessions were carried out remotely using a shared digital collaboration platform. Each team also had a facilitator and was connected to expert futurists for consultations throughout the process. For each think tank team, the sprint followed the six steps outlined in Fig. 1.

Briefly, the process involved six main exercises. The process can loosely be divided in a “divergent” thinking phase, i.e. a phase in which rich information is generated through brainstorming exercises, and a “convergent” thinking phase, i.e. one in which the rich information generated in the first phase is synthesized into more tangible outputs. Exercises involved in the divergent phase consisted in the identification of trends, the exploration of primary, secondary and tertiary consequences through futures wheels and the exploration of possible interactions between different trends and events through cross-impact analysis. The convergent phase involved writing rich narratives for scenarios, developing a roadmap to desirable futures through backcasting, and the consideration of implications for WHO at different levels. Throughout the process, work was performed as remote agile teams and emerging information was taken into consideration by teams in real time. Finally, the social, technological, economic, environmental and political (STEEP) lenses were applied in many exercises. The outputs of think tanks were distilled in a later phase of the process focused on integrating findings and broader backcasting for the ongoing COVID-19 response.

## 4 Reflections on the use of foresight for the new future

**Fig. 2:**
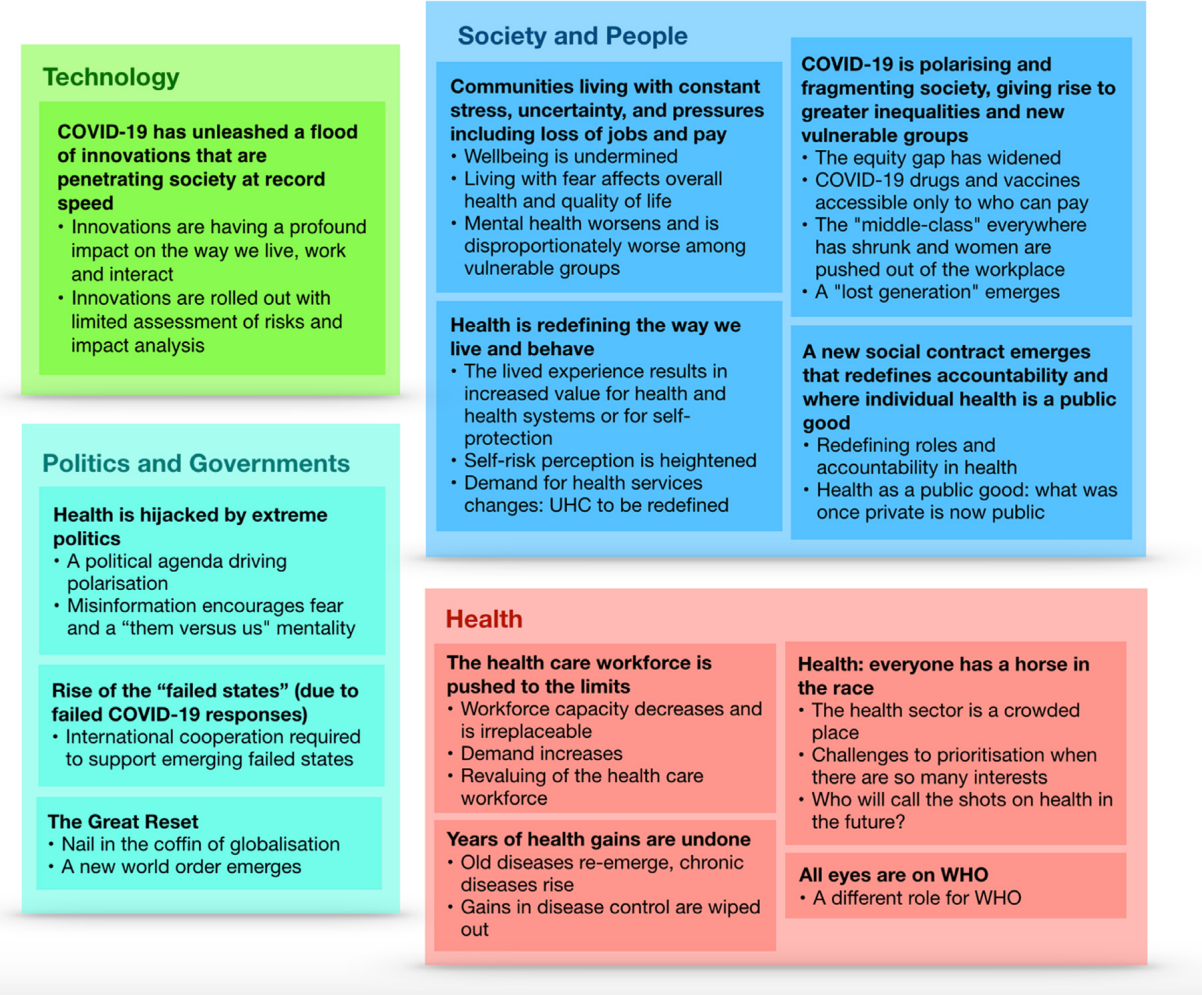
**The twelve dimensions of the new future.** Cross-cutting themes emerging from think tank groups were synthesized into twelve dimensions relating to society and people, health, politics and government, and technology.

Compared with scenarios developed by Inayatullah and Black [20], think tank scenarios took greater account of social, economic and political contexts and had more granularity and regional relevance. Each team used their expertise to build out aspects of these worlds, from the way that healthcare systems would operate to the risks felt by different vulnerable populations. Fig. 2 illustrates the twelve dimensions of the new future resulting from the synthesis of themes emerging from across think tanks. These relate to society and people, health, politics and governments, and technology. This synthesis of think tank outputs highlights the breadth and complexity of changes that could differentially emerge and coexist in different contexts depending on the prevailing scenario.

Habegger [27] stressed the importance of “strategic foresight that deliberately cuts across the traditional boundaries of policy areas and government departments”. The think tanks served as a forum to engage across departments and with external experts in a way that does not occur in routine operations. This enabled consideration of issues that may fall between WHO programs or ideas that sit outside the normal operations of WHO. Employing foresight methodologies also complemented conventional pandemic response mechanisms, as highlighted in the following sections.

### 4.1 Mapping complexity for a more holistic pandemic response

The divergence and convergence processes employed in foresight (Fig. 1) require mapping the complexity of possible futures before distilling learnings into scenarios and key actions. This allows for more perspectives and uncovers new issues, but also new ways to amplify existing issues. In the non-COVID mortality think tank, this allowed exploring different potential routes to non-COVID mortality, which led to the development of a framework for how mortality and morbidity in different disease categories could be affected as a result of COVID-19. Throughout the process, the group referred to lessons learned from the 2014–2016 Ebola epidemic, during which reduced access to treatments for malaria, HIV/AIDS and TB resulted in 10,600 estimated excess deaths in Sierra Leone, Liberia and Guinea compared to 11,300 deaths reported for Ebola in the same countries [28]. The team also continuously scanned for emerging signals of the pandemic's effects on different disease areas across the globe. Social, technological, economic, environmental and political factors related to the pandemic, explored throughout the process, were mapped as affecting different disease categories through intermediate health system effects. These health system effects were distilled into six categories: health seeking behaviour, service availability, health workforce, medicine and supplies, health funding and exposure to risk factors.

### 4.2 Highlighting emerging issues and paradigms for public health policy

Looking to new future scenarios also requires revisiting conventional definitions and paradigms used in public health and policy. For example, the increasing reliance on virtual platforms for healthcare delivery may result in the marginalization of populations with low access to technology. Access to the digital world may thus become an essential, and timely, dimension to add to conventional definitions of vulnerable groups. Geopolitical dynamics are seeing the added factor of COVID-19 medical supply availability and demand, exemplified by the idea of “face mask diplomacy”. Mental health, previously commonly limited to mental illness, emerged as a universally shared experience given the widespread increase in anxiety, fear and uncertainty. A new social contract is also cementing the individual's responsibility to the collective, resulting in increased emphasis on public and social health. The pandemic is thus acting as a magnifying glass for existing issues, as evidenced from increased discussion about business responsibilities to their employees and government responsibilities towards their citizens. The re-negotiation of this social contract may stem into polarized effects on the social fabric.

### 4.3 Moving towards a more resilient pandemic response by considering the full range of what is possible

Unlike predictions that collapse futures into most probable outcomes, which lose the richness of possibilities, foresight accounts for the possibility of multiple futures, including the least probable ones. Mapping these futures requires exploring uncertainties rather than ignoring implications of what is not yet predictable. It also considers possible *wild cards* (high impact, low probability events): what if a cyclone hit the region during the pandemic? What if a secondary epidemic occurred due to a natural disaster? What if a country experienced radical political turmoil or a coup d’état? The importance of accounting for what is possible, and not just what can be assigned a probability, was highlighted by the realization of at least two wild card events in the process of the think tank sprints: the hacking of WHO systems and severe tropical cyclone Harold hitting Vanuatu, Fiji, Tonga and the Solomon Islands.

### 4.4 Identifying strategic opportunities

Backcasting from the new future to the implications for today enabled the consideration of possible strategic entry points for WHO. Opportunities could arise by leveraging some of the positive trends that may be boosted by the pandemic. For example, universal health coverage (UHC), one of the targets under the Sustainable Development Goals (SDGs) as well as one of WHO's strategic priorities, is likely to see revitalized commitment, with the pandemic bringing increasing social value of health. The pandemic may also provide new momentum in consolidating the often-divergent definitions of essential services. The universal increase in stress stemming from the pandemic has been identified as a driver for a renaissance of efforts towards mental health as a fundamental aspect of health and shared human experience. Reliance on digital health and innovative healthcare delivery models could be an opportunity to catalyze capacity building, responsible regulation and sustainable planning of digital health technologies and innovations, while implementing standards and preventing fragmentation and conflicts of interest. This process helps WHO draw out ideas for initiatives that align with its organizational values. It also creates a model for how those might emerge, and therefore the signals to look for in order to make the most of the windows of opportunity.

## 5 Conclusions

In conclusion, the implementation of foresight approaches in strategic planning in the context of a pandemic offers several benefits when overlaid to conventional pandemic response mechanisms. By engaging with the same information using different models and creating rich future scenarios that consider predictable trends, but also areas of uncertainty and divergence, potential shocks to the system and complex interactions, a more future-proof strategy can be devised. Given the diversity and complexity of country contexts and systems, this approach is particularly helpful in developing more tailored responses. Different futures may coexist across and within different countries. The process further serves as a forum to engage external expertise and bring together diverse skillsets from within an organization.

Findings from think tanks were presented to different internal stakeholders as well as through a webinar involving WHO country offices, thus sparking important conversations and new ways of thinking early on in the regional pandemic response efforts. The results were also presented at the fourth meeting of the Technical Advisory Group on UHC in the Western Pacific Region, a meeting having 195 participants, as a way of getting Member States and experts to think about how to re-build and re-fresh UHC in the post COVID-19 era.

As any process, the setup described has several limitations. The process was necessarily limited by the expertise and number of participants. The intensive pace and unusually short period of time may affect the quality, timeliness and availability of information. The shape of the epidemic curve, country-level action, and information about the biology of the virus were changing on a daily basis. The exercise was therefore necessarily qualitative, not having the benefit of data on several issues, including longitudinal data about people and communities. The level of global uncertainty was also unusually high, making even planning for an 18-month horizon difficult. This is why it is important to revisit early foresight outputs in an iterative manner, as exemplified by one of the effective foresight rules “hold strong opinions weakly” outlined by Saffo [29]. Shortening the process and delivering it in a virtual environment also constrained methodology design. For example, archetype scenarios were provided for participants to further develop, instead of scenarios being developed based on the dynamics the teams were most interested in exploring.

Considerations in the implementation of foresight emerge in the specific context of a multi-lateral agency like WHO. Understanding policy-making systems and dynamics (through governance mapping in the process) may be beneficial in future iterations, insofar as it helps WHO anticipate potential scenarios in different countries and therefore frame advice in relation to culture and politics. Further, the constitution, functions and operations of WHO pose constraints on the organization's ability to work differently. For instance, donor financing tied to vertical programs limits flexibility and agility, its workforce and culture are heavily medically and epidemiologically oriented, and its commitment to neutrality is core to both being part of the UN system and to the trust that countries establish with the organization. Further limitations are posed by individual time constraints, competing priorities amid the pandemic, and the necessity for participants to engage remotely through online platforms, a format that inevitably loses richness compared to an in-person workshop format.

Despite these limitations, the usefulness of the process in informing a long-term strategic plan for WPRO to meet the needs of the possible new futures for the Western Pacific region suggests similar benefits may be reaped by engaging stakeholders with a range of expertise through dialogues across government sectors and within countries. Its effectiveness further extends beyond post-pandemic planning to other highly prevalent health policy issues on longer time horizons including non-communicable diseases and ageing.

## Author Contributions

Maria Isabella Gariboldi and Vivian Lin wrote the article. Jessica Bland provided foresight expertise for the article. Amy Cawthorne ideated and led the foresight think tank project, with coordination and content support from Maria Isabella Gariboldi and Mallika Auplish. Amy Cawthorne also led the synthesis process.

## Declaration of Interests

The authors have no conflicts of interest.
